# Alteration in the mRNA expression profile of the autophagy-related mTOR pathway in schizophrenia patients treated with olanzapine

**DOI:** 10.1186/s12888-021-03394-w

**Published:** 2021-08-04

**Authors:** Fengwei Cui, Shuguang Gu, Yue Gu, Jiajun Yin, Chunxia Fang, Liang Liu

**Affiliations:** 1 Department of Geriatric Psychiatry, Wuxi Mental Health Center, Nanjing Medical University, Wuxi, 214151 Jiangsu China; 2 The First Clinical Medical College, Nanjing Medical University, Nanjing, 211166 Jiangsu China; 3 Combined TCM & Western Medicine Department, Wuxi Mental Health Center, Nanjing Medical University, Wuxi, 214151 Jiangsu China

**Keywords:** Schizophrenia, mTOR pathway, DEPTOR, mRNA expression, Olanzapine

## Abstract

**Background:**

The mammalian target of rapamycin protein (mTOR) signaling pathway is involved in the pathogenesis of schizophrenia and the mechanism of extrapyramidal adverse reactions to antipsychotic drugs, which might be mediated by an mTOR-dependent autophagy impairment. This study aimed to examine the expression of mTOR pathway genes in patients with schizophrenia treated with olanzapine, which is considered an mTOR inhibitor and autophagy inducer.

**Methods:**

Thirty-two patients with acute schizophrenia who had been treated with olanzapine for four weeks (average dose 14.24 ± 4.35 mg/d) and 32 healthy volunteers were recruited. Before and after olanzapine treatment, the Positive and Negative Syndrome Scale (PANSS) was used to evaluate the symptoms of patients with schizophrenia, and the mRNA expression levels of mTOR pathway-related genes, including MTOR, RICTOR, RAPTOR, and DEPTOR, were detected in fasting venous blood samples from all subjects using real-time quantitative PCR.

**Results:**

The MTOR and RICTOR mRNA expression levels in patients with acute schizophrenia were significantly decreased compared with those of healthy controls and further significantly decreased after four weeks of olanzapine treatment. The DEPTOR mRNA expression levels in patients with acute schizophrenia were not significantly different from those of healthy controls but were significantly increased after treatment. The expression levels of the RAPTOR mRNA were not significantly different among the three groups. The pairwise correlations of MTOR, DEPTOR, RAPTOR, and RICTOR mRNA expression levels in patients with acute schizophrenia and healthy controls were significant. After olanzapine treatment, the correlations between the expression levels of the DEPTOR and MTOR mRNAs and between the DEPTOR and RICTOR mRNAs disappeared.

**Conclusions:**

Abnormalities in the mTOR pathway, especially DEPTOR and mTORC2, might play important roles in the autophagy mechanism underlying the pathophysiology of schizophrenia and effects of olanzapine treatment.

## Background

Schizophrenia is a serious chronic mental disorder, and its prevalence rate is approximately 1% of the global population [1]. The pathogenesis of schizophrenia is not completely clear, and no reliable objective biological indicators for adjuvant diagnosis and treatment are available. Recent research has indicated that the mammalian target of rapamycin protein (mTOR) pathway may be involved in schizophrenia pathogenesis [2].

mTOR is a highly conserved serine/threonine kinase with a molecular weight of 289 kDa [3]. mTOR, regulatory-associated protein of mTOR (RAPTOR), and DEP domain-containing MTOR-interacting protein (DEPTOR) form the mTORC1 complex, which is mainly involved in cell growth, apoptosis, energy metabolism, and autophagy; meanwhile, mTOR, DEPTOR, and rapamycin-insensitive companion of mTOR (RICTOR) form the mTORC2 complex, which is mainly involved in the construction and maintenance of cytoskeletal proteins [4]. The main upstream signaling pathways of mTOR are the PI3K/Akt pathway and the AMPK pathway. The major effector target proteins of the mTOR downstream pathway are 4E-BP1 and S6K1, which are involved in protein translation initiation, gene transcription, and cell cycle regulation [5].

Alterations in the mTOR pathway might contribute to the regulation of synaptic function. mTOR mainly regulates the rate-limiting step in the initial local translation of a particular gene upon synaptic activation and induces de novo dendritic protein synthesis, which allows the long-term maintenance of functional changes in the synapse [6–8]. Thus, synapses can process and store the received information [9]. Calabrese et al. proposed that mTOR alterations might contribute to the synaptic dysfunctions that characterize several psychiatric diseases and that the mTOR pathway might represent a potential target for pharmacological intervention [10]. As shown in the study by Rail et al., DRD1-Cre mTOR-conditional knockout male mice show hyperpolarization and a coincident decrease in the distal spine density in striatal direct pathway striatal projection neurons through an intricate mechanism involving RhoA (Ras homolog family member A) and culminating in Kv1.1 (potassium voltage-gated channel subfamily A member 1) hyperfunction, finally leading to decreased spontaneous locomotion, impaired social interaction, and decreased marble-burying behavior [11]. Lin et al. also reported that elevated mTORC1-S6K1 (ribosomal S6 kinase 1, S6K1) signaling occludes dynamic DRD1 signaling downstream of DARPP-32 (protein phosphatase 1, regulatory inhibitor subunit 1B) and blocks multiple DRD1 responses, including dynamic gene expression, DRD1-dependent corticostriatal plasticity, and DRD1 behavioral responses, such as sociability, while the levels of candidate biomarkers of mTORC1-DARPP-32 occlusion are increased in the brains of human subjects with a disease in association with elevated mTORC1-S6K1 levels [12].

The mTOR signaling pathway might be involved in the pathogenesis of schizophrenia. Neurobiochemical studies have inferred that the mTOR signaling pathway is involved in the downstream signal transduction mechanism of genes related to schizophrenia, such as dopamine receptor D2 (DRD2) [13], disrupted in schizophrenia 1 (DISC1) [14], brain-derived neurotrophic factor (BDNF) [15], N-methyl-D-aspartate receptor (NMDAR) [16], 5-HT receptors (5-HTRs) [17], and Reelin [18]. Mice with disordered mTORC2 signaling in the brain exhibit changes in striatal DA-dependent behavior, such as increased basal ganglia-related activities and stereotypy counts, as well as a significant enhancement of the psychomotor effects of amphetamine [19].

The mTOR signaling pathway plays a role in the mechanism of extrapyramidal adverse reactions to antipsychotic drugs. A gene expression microarray analysis of patients with first-episode schizophrenia before and after treatment with risperidone or paliperidone showed that the mTOR pathway was involved in extrapyramidal system reactions [20, 21]. The interaction of four single nucleotide polymorphism (SNP) loci, rs1130214 (AKT1), rs456998 (FCHSD1), rs7211818 (RAPTOR), and rs1053639 (DDIT4), was reported to predict the extrapyramidal response of patients with schizophrenia after using antipsychotic drugs. The accuracy of the prediction was 85–88% in 243 patients with schizophrenia treated with risperidone or other antipsychotic drugs [22].

In summary, the mTOR signaling pathway is involved in the downstream signal transduction mechanism of genes related to schizophrenia, in alterations in synaptic plasticity and cognitive functions, and in the mechanism of extrapyramidal adverse reactions to antipsychotic drugs. However, to date, little is known about the gene expression profile of the mTOR pathway in patients with schizophrenia. This study examined the expression levels of mTOR pathway genes in patients with schizophrenia before and after olanzapine treatment, which is very important to further understand the pathological changes in the mTOR pathway related to schizophrenia.

## Methods

### Participants

Forty-five inpatients with acute schizophrenia episodes were recruited from September 2017 to June 2018 at Wuxi Mental Health Center of Nanjing Medical University as the case group. Three patients were excluded later because of a revised diagnosis, and 10 patients were lost to follow-up because they did not complete the evaluation and blood collection in the fourth weekend. At the time of admission, one deputy chief psychiatrist made identical diagnoses according to the diagnostic criteria for schizophrenia in the Diagnostic and Statistical Manual of Mental Illness, 4th Edition (DSM-IV) without other Axis 1 diagnoses, and the total PANSS score for each patient was > 90. Forty-six healthy volunteers in the healthy control group were recruited from people undergoing health examinations at Wuxi Tongren Rehabilitation Hospital. All subjects included in the study were aged between 18 and 60, Han nationality; none of them had blood transfusion treatment, substance abuse history, physical diseases, and received treatment of antipsychotics, antidepressants, emotional stabilizers, electroconvulsive therapy in the past three months; none of the female subjects were in lactating, pregnant, and menstruating at the time of enrollment.

### Olanzapine therapeutic regimen

Enrolled patients with schizophrenia received olanzapine as a single-drug therapy. The initial dose of olanzapine was 5 mg/d, and the therapeutic dose was increased to 10–20 mg/d after one week. The average dose of olanzapine across all the patients was 14.24 ± 4.35 mg/d, and the observation period was 4 weeks. The treatment was not combined with other antipsychotics, antidepressants, emotional stabilizers, sedatives, or electroconvulsive therapy for any of the patients in the study.

### Clinical data collection

On the day of enrollment, clinical data were collected from patients with schizophrenia by one psychiatric doctor who had passed the consistency training for a PANSS assessor at the Sixth Hospital of Peking University. The psychiatric symptoms of all patients with schizophrenia were assessed at baseline and the fourth weekend of drug treatment.

### Experimental process of mRNA expression level detection in blood samples

The process of fasting venous blood extraction, total RNA extraction, reverse transcription reaction, and real-time quantitative PCR referred to our previous paper [23]. The primers for PCR reaction were listed in Table 1 with GAPDH as the housekeeping gene. Delta-delta Ct was used to detect the relative mRNA expression [24].

**Table 1:** Primer sequence and PCR product length of housekeeping gene and target genes

Gene	Primer types	Primer sequence	Tm value(°C)	PCR product length (bp)
GAPDH	Forward	GGCCTCCAAGGAGTAAGACC	60.07	122
	Reverse	AGGGGAGATTCAGTGTGGTG	59.96	
MTOR	Forward	AGCCGGAATGAGGAAACC	60.01	232
	Reverse	CAAATCTGCCAATTCGGG	60.00	
DEPTOR	Forward	ATTGTTGGTGACGCGGTT	59.97	137
	Reverse	AGCCCGTTGACAGAGACG	59.98	
RICTOR	Forward	AAGGCCAAACAGCTCACG	59.98	230
	Reverse	ACTCCATGAGGGTGGCAA	60.05	
RAPTOR	Forward	CAGGACTTGCTGGTGGCT	59.98	84
	Reverse	GCTGACGGGAGTGCAGTT	59.99	

### Statistical analyses

Data collation and analysis were performed with the SPSS 19.0 statistical software package. The sex and age data were compared between the case group and control group with the *t* test and *χ*^2^ test. The extreme values of mRNA expression levels (no more than 8 values among 384 values for the four target genes in three groups) were determined by constructing a box chart based on the quartile method and quartile range, and handled with the winsorizing method. After the Kolmogorov-Smirnov normal distribution test and Levene variance homogeneity test, one-way ANOVA was used to compare the expression levels of the target genes of three groups, and Tamhane’s T2 test was used as the post hoc test. The data are presented as the means and standard deviations ($\overline{x}$ + *SD*), and the test level was α = 0.05 bilaterally.

## Results

### No significant difference in demographic and clinical data

Thirty-two patients with schizophrenia completed the study, including 16 female patients and 16 male patients, with an average age of 37.53 ± 10.84 years. After admission, their average dose of olanzapine was 14.24 ± 4.35 mg/d. The 46 healthy volunteers in the healthy control group included 19 females and 27 males, with an average age of 36.78 ± 10.96 years. No significant differences in sex, age, marital status, or years of education were observed between the case group and control group (*p* > 0.05; Table 2).

**Table 2:** Clinical data between case group and control group

	Case group (***n*** = 45)	Control group (***n*** = 46)	***t*** /***χ***^**2**^ test (p)
Age, Mean (SD)	38.20 (11.72)	36.78 (10.96)	0.596 (0.553)
Male, N (%)	22 (48.9)	27 (58.7)	0.530 (0.467)
Education, Mean (SD)	10.56 (3.71)	11.38 (4.69)	0.924 (0.358)
Married, N (%)	20 (44.4)	24 (52.1)	0.114 (0.736)
Onset age, Mean (SD)	31.22 (11.26)	NA	NA
First-episode patients, N (%)	15 (33.3)	NA	NA
Disease duration (years), Mean (SD)	2.80 (1.91)	NA	NA
Olanzapine dose (mg/d), Mean (SD)	14.24 (4.35)	NA	NA
PANSS score, Mean (SD)			
Before treatment	115.84 (15.64)	NA	NA
After treatment	76.38 (14.72)	NA	NA

Note: The data of sex and marital status were compared with *χ*^2^ test, and the data of age and years of education was compared with t test. The data of sex, age, marital status and years of education had no significant difference between the case group and control group (p>0.05)

### Divergent mTOR pathway gene expression levels in the three groups

**Fig. 1:**
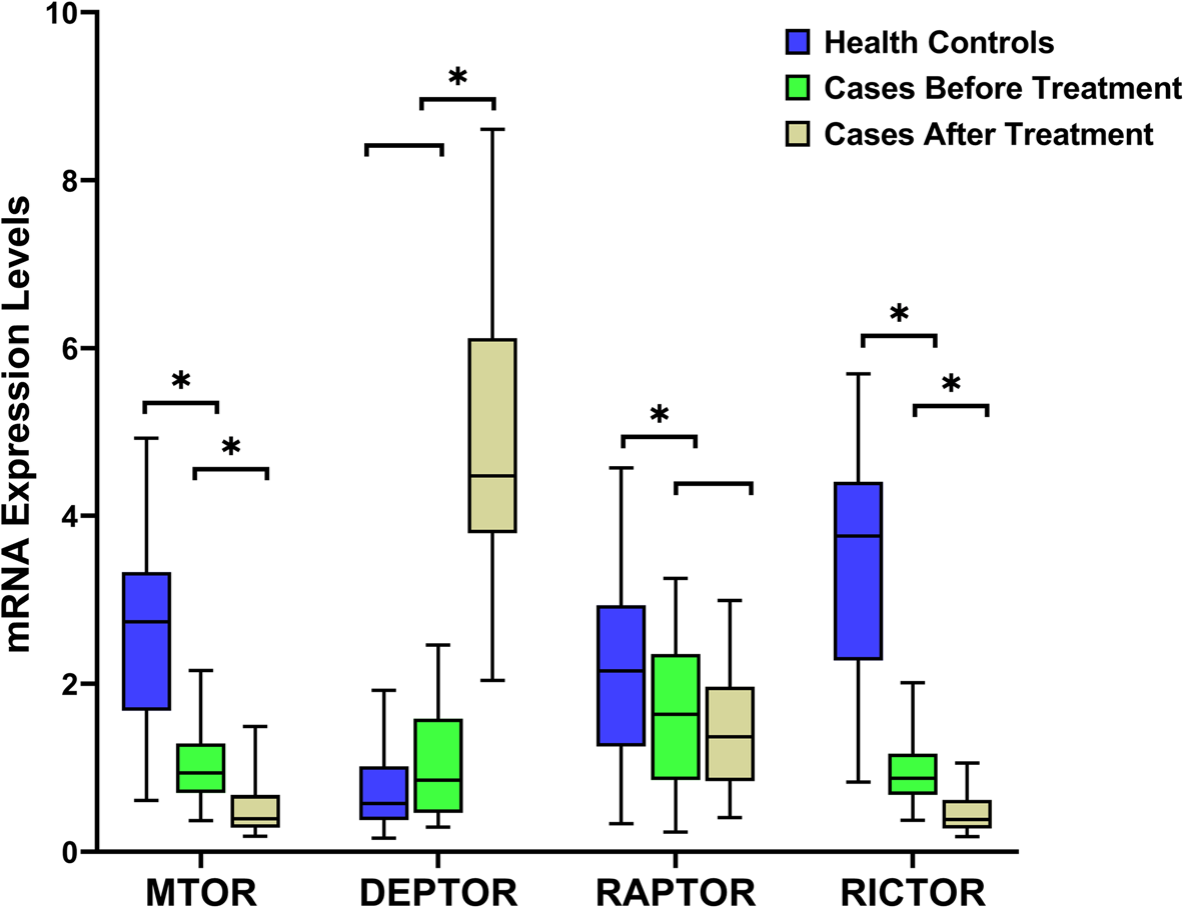
mTOR pathway genes expression levels in the control group and case group before and after olanzapine treatment. MTOR and RICTOR mRNA expression levels of acute schizophrenia patients significantly decreased than them of healthy controls, and furtherly significantly decreased after 4 weeks of olanzapine treatment. While DEPTOR mRNA expression levels of acute schizophrenia patients had no significant difference with them of healthy controls, but significantly increased after 4 weeks of olanzapine treatment. The mRNA expression levels of RAPTOR had no significant difference in three groups

The comparison of the mRNA expression levels of mTOR pathway genes revealed significantly lower MTOR, RAPTOR, and RICTOR mRNA expression levels in the case group before olanzapine treatment than in the control group, while no significant difference in DEPTOR mRNA levels was observed between groups. After olanzapine treatment, the DEPTOR mRNA expression levels increased significantly in the case group, with no significant differences in the other three target genes (Table 3 and Fig. 1).

**Table 3:** Comparison of mTOR pathway genes expression levels in three groups

	Case group		Control group	*F*	*p*	Multiple comparisons		
	Baseline (n = 45)	After treatment (*n* = 32)	(n = 46)			MD^a^	MD^b^	MD^c^
MTOR^a,b,c^	1.08 ± 0.59	0.51 ± 0.30	2.41 ± 1.19	57.03	0.000	0.568	1.338	1.906
DEPTOR^a,c^	1.00 ± 0.61	4.92 ± 1.75	0.75 ± 0.52	188.445	0.000	3.913	0.251	4.164
RAPTOR ^c^	1.79 ± 1.07	1.60 ± 1.13	2.27 ± 1.28	3.70	0.028	0.191	0.492	0.683
RICTOR^a,b,c^	1.06 ± 0.58	0.49 ± 0.29	3.18 ± 1.48	86.80	0.000	0.564	2.128	2.692

Note: Levene variance homogeneity test showed that the expression level data of four target genes were not uniform. One way ANOVA was used to compare the expression levels of target genes in 3 groups, and Tamhane’s T2 test was used to multiple comparisons

^a^ Comparison of mRNA expression levels of target genes in case group before and after treatment had significant difference (*p* < 0.05);

^b^ Comparison of mRNA expression levels of target genes between control group and case group before treatment had significant difference (*p* < 0.05);

^c^ Comparison of mRNA expression levels of target genes between control group and case group after treatment had significant difference (*p* < 0.05)

### Changes in the correlations of mTOR pathway gene expression levels after olanzapine treatment

**Fig. 2:**
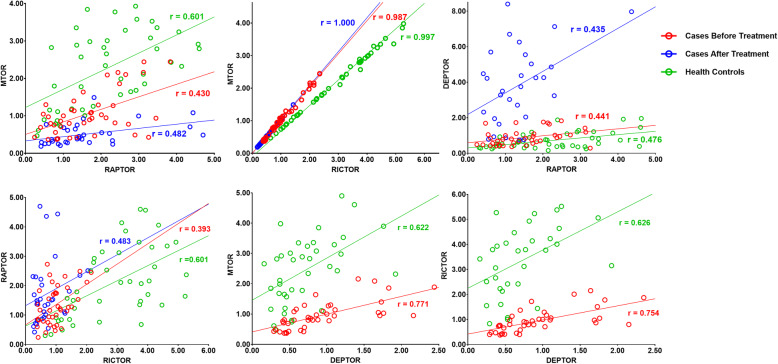
Correlation alteration of mRNA expression levels of mTOR pathway genes. MTOR, DEPTOR, RAPTOR, and RICTOR mRNA expression levels in patients with acute schizophrenia and healthy controls were significantly pairwise correlated in all three groups, and the MTOR and RICTOR mRNA expression levels had the highest correlation (*r* = 0.987–1.000). However, after 4 weeks of olanzapine treatment, the pairwise correlations of mRNA expression levels disappeared between DEPTOR and MTOR, and also between DEPTOR and RICTOR

MTOR, DEPTOR, RAPTOR, and RICTOR gene expression levels in case group before olanzapine treatment and the control group were significantly pairwise correlated, with the MTOR and RICTOR gene expression levels showing the highest correlation (*r* = 0.987–1.000). However, after 4 weeks of olanzapine treatment, the correlations between DEPTOR and MTOR mRNA expression, and between DEPTOR and RICTOR mRNA expression disappeared (Fig. 2).

### No correlations between mTOR pathway gene expression levels, and clinical data

No correlations were observed between the four target genes and demographic or clinical data, such as sex, age, marital status, education level, first episode or recurrence, disease course, or PANSS score, in the healthy control group or case group before or after treatment.

## Discussion

The mTOR signaling pathway is present in almost all peripheral tissues and the central nervous system and is involved in regulating protein synthesis, mitochondrial biogenesis, cell proliferation, cell survival, cell death [25, 26], and synaptic plasticity [15]. Abnormalities in the mTOR pathway have attracted increasing attention and have been identified in many diseases, including cancer [27], obesity [28], type II diabetes mellitus [29], neurological and psychiatric diseases [30], neurodegeneration, and brain tumors. Recent studies have shown that many psychiatric drugs, including mood stabilizers and neurorelaxants, which are also autophagy-inducing factors, regulate autophagy and exert a therapeutic effect on the mTOR pathway [31, 32].

### Alterations in the expression of mTOR pathway genes in patients with schizophrenia treated with olanzapine

To date, relatively few studies have examined the relationship between the expression levels of the mTOR pathway and the pathogenesis of schizophrenia. They have mostly been preclinical studies, with few studies involving patients with schizophrenia. In the present study, patients with acute schizophrenic were recruited to study the expression levels of the MTOR, DEPTOR, RAPTOR, and RICTOR genes in the mTOR pathway before and after olanzapine treatment. Before olanzapine treatment, the MTOR, RAPTOR, and RICTOR mRNAs were expressed at significantly lower levels in the case group than in the control group, while DEPTOR mRNA expression levels showed no significant differences between the two groups. After olanzapine treatment, the DEPTOR mRNA expression levels significantly increased in the case group, with no significant differences detected in the other three target genes.

Few studies have researched MTOR gene expression in patients with mental disorders. Mostaid et al. found that mTOR mRNA expression levels were negatively correlated with the duration of illness in patients with treatment-resistant schizophrenia, and clozapine exposure decreased mTOR mRNA expression levels in an in vitro culture of PBMCs from patients with treatment-resistant schizophrenia [33]. Machado-Vieira et al. found decreased mTOR mRNA expression levels in 25 unmedicated depressed individuals with bipolar disorder, which showed no significant change after 6 weeks of lithium therapy [34]. Dong et al. suggested that prenatal stress induces decreased mTOR mRNA levels, which may be associated with anxiety-like and alcohol drinking behaviors in adulthood [35]. Based on these studies, the MTOR gene may be abnormally expressed in patients with schizophrenia and other psychiatric disorders. Our results revealed significantly lower MTOR mRNA expression levels in patients with acute schizophrenia before treatment than in healthy controls, and the levels did not change significantly after olanzapine treatment.

DEPTOR has been reported to be an endogenous regulator of mechanistic target of rapamycin complex 1 (mTORC1) and mTORC2. DEPTOR is widely expressed in regions ranging from the forebrain to the hindbrain, including the hippocampus, the mediobasal hypothalamus, and the circumventricular organs (CVOs) [36]. Relatively few studies have assessed DEPTOR expression in patients with mental disorders and no reports have documented its expression in patients with schizophrenia. Fabbri and Serretti used the Systematic Treatment Enhancement Program for Bipolar Disorder (STEP-BD) genome-wide dataset to investigate the genetic predictors of long-term treatment outcomes and found that the DEPTOR gene, which is susceptible to antidepressant action, may affect the long-term treatment outcome of patients with bipolar disorder [37]. Davies et al. documented a reduction in DEPTOR protein levels in the precentral gyrus, postcentral gyrus, and occipital lobe of patients with Alzheimer’s disease (AD), as well as a reduction in DEPTOR expression in patients with late-onset AD compared to individuals with early-onset familial AD [38]. In our study, DEPTOR mRNA expression did not differ significantly between healthy controls and patients with acute schizophrenia before treatment, but after 4 weeks of olanzapine treatment, the DEPTOR expression level increased significantly in patients with schizophrenia.

RAPTOR is an important component of mTORC1 and a regulator of mTOR. RAPTOR knockout mice show decreased body weight, brain weight, and cortical thickness compared to 7-week-old wild-type mice [39]. Research on RAPTOR in mental disorders has focused on the predictive function of mTOR pathway-related genes in antipsychotic-induced extrapyramidal symptoms (EPS). Mas et al. analyzed gene–gene interactions among nine genes related to the mTOR pathway to develop genetic predictors of the appearance of EPS and identified a four-way interaction among rs1130214 (AKT1), rs456998 (FCHSD1), rs7211818 (Raptor), and rs1053639 (DDIT4) that correctly predicted AP-induced EPS in 97 of the 114 patients (85% accuracy). Then, the authors validated the predictive power of the four-way interaction in two independent cohorts and reported 86 and 88% accuracy, respectively [22]. Boloc et al. developed a pharmacogenetic predictor of antipsychotic-induced EPS based on two SNPs in the AKT1 gene (rs33925946 and rs1130214) and two SNPs in the RAPTOR gene (rs3476568 and rs9915667) in 131 inpatients with schizophrenia treated with risperidone. Their prediction model achieved 66% accuracy for antipsychotic-induced EPS in the discovery cohort and showed similar performance in replications of the schizophrenia cohort treatment with risperidone or other antipsychotics [40]. In the present study, significantly lower RAPTOR mRNA expression levels were detected in patients with acute schizophrenia before treatment than in healthy controls, and the levels did not change significantly after olanzapine treatment.

RICTOR is a component of mTORC2. Experiments with RICTOR KO animals have identified an important role for this gene in the pathogenesis of schizophrenia. Dadalko et al. found that neuron-specific RICTOR knockout mice exhibited altered striatal DA-dependent behaviors, such as increased basal locomotion and stereotypy counts and an exaggerated response to the psychomotor effects of amphetamine [19]. According to Siuta et al., neuronal Rictor knockout mice show impairments in neuronal Akt Ser473 phosphorylation, prepulse inhibition deficits, hypodopaminergia in the rostral cortex, an increase in NE transporter expression and function, and schizophrenia-like behaviors [41]. Moreover, RICTOR is also associated with the pathological mechanisms of other mental disorders. Miyata et al. used ovariectomized (OVX) mice exposed to chronic mild stress to simulate depression during menopause and conducted studies of genome-wide gene expression in both the medial prefrontal cortex and blood cells. RICTOR was the top-ranked regulator associated with the OVX-induced alterations in gene expression in both tissues [42]. Eriguchi et al. used exome sequencing to identify novel risk loci for sporadic Tourette syndrome cases and found that rs140964083 (RICTOR) was a novel candidate factor for Tourette syndrome etiology [43]. In the present study, the RICTOR mRNA expression level was significantly lower in patients with acute schizophrenia before treatment than in healthy controls and did not change significantly after olanzapine treatment.

### Alterations in correlations between mTOR pathway gene expression levels indicated the dysfunction of DEPTOR and the mTORC2 complex

As shown in the present study, MTOR, DEPTOR, RAPTOR, and RICTOR mRNA expression levels exhibited significant pairwise correlations in patients with acute schizophrenia and the normal control group, and MTOR pathway genes might interact and coordinate as a whole to exert their biological functions. However, after 4 weeks of olanzapine treatment, the pairwise correlations between DEPTOR and MTOR mRNA expression and between DEPTOR and RICTOR mRNA expression disappeared. MTOR, DEPTOR, and RICTOR are the key components of the mTORC2 complex. mTORC1 is a key inhibitor of autophagy, yet the function of mTORC2 in autophagy is controversial. However, more papers have recently begun to focus on the role of mTORC2 in autophagy mechanisms. Bernard et al. identified that reactive oxygen species (ROS) are one of the central inducers of mTORC2 activation during chronic autophagy [44]. Aspernig et al. found that the inactivation of mTORC2-SGK-1 (serum/glucocorticoid regulated kinase 1, SGK-1) signaling impairs mitochondrial homeostasis and triggers the increased release of mitochondria-derived reactive oxygen species (mtROS) to induce autophagy [45]. In the study by Lampada et al., genetic inhibition of mTORC2 and pharmacological inhibition of both mTORC1/2 led to decreased phosphorylation of c-MET, one of the receptor tyrosine kinases, in autophagy-proficient but not autophagy-impaired cells [46]. Olanzapine, an autophagy activator, protects neurons from fatal mitochondrial damage [47]. Therefore, these findings might suggest that olanzapine may modulation the expression of the DEPTOR mRNA, the formation of the mTOR complex, and even neuronal autophagy.

## Conclusions

In the present study, the mRNA expression levels of MTOR, RAPTOR, and RICTOR were significantly lower in the case group before olanzapine treatment than in the control group, while DEPTOR mRNA expression levels showed no significant difference between the two groups. After olanzapine treatment, the DEPTOR mRNA expression levels were significantly increased in the case group, with no significant differences in the expression of the other three target genes. Additionally, mTOR pathway genes might interact and coordinate as a whole to exert their biological functions in healthy controls and patients with acute schizophrenia. However, after olanzapine treatment, the pairwise correlations between the mRNA expression levels of DEPTOR and MTOR and of DEPTOR and RICTOR disappeared. We inferred that olanzapine may modulate the expression of the DEPTOR mRNA and the formation of mTORC2. To date, the exact function of the DEPTOR gene in schizophrenia has not been completely elucidated. The role of the mTOR pathway in the curative effect of and adverse reactions to antipsychotic drugs has also been mentioned in several studies, as described above.

The limitations of this paper included small sample size and high dropout rate of patients. This research controlled the drug factor, excluding physical therapy, but did not consider the effects of psychological factors, environmental factors, and social support factors. Considering the difference in individual drug metabolism, the blood olanzapine concentrations should be the indicator for drug-controlling factors instead of its oral dose to ensure that the research is more accurate. Meanwhile, there is uncertainty in the consistency of target genes expression levels in peripheral blood and central nervous system. Nevertheless, this study is a promotion to understand the role of mTOR pathway in autophagy mechanism of patients with schizophrenia, and to explore whether these genes are potential biomarkers for the diagnosis of and determination of therapeutic efficacy in patients with schizophrenia.

## Data Availability

Data of this study are not publicity available as being a part of a broader project, which data are still analyzing, but are available from the corresponding author on reasonable request.

## Abbreviations

4E-BP1: Eukaryotic initiation factor 4E binding protein 1
5-HTRs: 5-HT receptors
AD: Alzheimer’s disease
AKT: Serine/threonine kinase
AMPK: Protein kinase AMP-activated catalytic subunit alpha 1
BDNF: Brain-derived neurotrophic factor
c-MET: MET proto-oncogene, receptor tyrosine kinase
CVOs: Circumventricular organs
DARPP-32: Protein phosphatase 1, regulatory inhibitor subunit 1B
DDIT4: DNA damage inducible transcript 4
DEPTOR: DEP domain-containing MTOR-interacting protein
DISC1: Disrupted in schizophrenia 1
DRD1: Dopamine receptor D1
DRD2: Dopamine receptor D2
DSM-IV: Diagnostic and Statistical Manual of Mental Illness, 4th Edition
EPS: Extrapyramidal symptoms
FCHSD1: FCH and double SH3 domains 1
Kv1.1: Potassium voltage-gated channel subfamily A member 1
mTOR: Mammalian rapamycin target protein
mTORC1: Mechanistic target of rapamycin complex 1
mTORC2: Mechanistic target of rapamycin complex 2
mtROS: Mitochondria-derived reactive oxygen species
NMDAR: N-methyl-d-aspartate receptor
OVX: Ovariectomized
PANSS: Positive and Negative Syndrome Scale
PI3K: Phosphatidylinositol 3-kinase
RAPTOR: Regulatory-associated protein of mTOR
RhoA: Ras homolog family member A
RICTOR: Rapamycin-insensitive companion of mTOR
ROS: Reactive oxygen species
S6K1: Ribosomal protein S6 kinase
SGK-1: Serum/glucocorticoid regulated kinase 1
SNP: Single nucleotide polymorphism
